# Clinicopathological Study of Hodgkin Lymphoma in Relation to Programmed Death-Ligand 1 (PD-L1) Expression and Epstein-Barr Virus (EBV) Status

**DOI:** 10.7759/cureus.98152

**Published:** 2025-11-30

**Authors:** Arifa B Laskar, Jaya Mishra, Yookarin Khonglah, Vikas K Jagtap

**Affiliations:** 1 Pathology, North Eastern Indira Gandhi Regional Institute of Health and Medical Sciences (NEIGRIHMS), Shillong, IND; 2 Radiation Oncology, North Eastern Indira Gandhi Regional Institute of Health and Medical Sciences (NEIGRIHMS), Shillong, IND

**Keywords:** ebv, hodgkin lymphoma, lmp-1, pdl-1, therapy

## Abstract

Introduction: T-cell anergy leads to upregulation and activation of programmed death-ligand 1 (PD-L1) expression, contributing to immune tolerance and the evasion of immune surveillance. Many cancers exploit this by expressing PD-L1 in neoplastic or non-neoplastic tumor microenvironment cells. Hodgkin lymphoma (HL) is notable for high PD-L1 expression, often associated with Epstein-Barr virus (EBV) in tumor cells. New therapies target this pathway. EBV gene products, like latent membrane protein-1 (LMP-1), can increase PD-L1 expression. Our study examined PD-L1 expression in Hodgkin and Reed-Sternberg (HRS) cells in HL patients, comparing it to EBV presence and clinical data.

Objective: The objective of this study is to study the clinicopathological characteristics of HL in relation to PD-L1 expression and EBV LMP-1 status.

Materials and methods: This ambispective study examined lymph node excision samples from 40 HL patients diagnosed between January 2014 and January 2024. Using immunohistochemistry, we evaluated PD-L1 presence in HRS cells, assessing the intensity and frequency of PD-L1 expression. We also detected EBV using the LMP-1 antibody. A non-parametric chi-square test was used for statistical analysis.

Results: A total of 82.5% (33/40) of cases had PD-L1 expression in HRS cells, with 65% (26/40) showing LMP-1 expression. LMP-1 expression shows a significant correlation with B symptoms and the histological subtypes of HL (p-value < 0.05). Similarly, PD-L1 expression is significantly correlated with B symptoms. Notably, 84.6% (22/26) of cases positive for LMP-1 also exhibit PD-L1 expression. However, EBV positivity was statistically significant when considering different histological subtypes.

Conclusion: PD-L1 and LMP-1 positivity are linked to B symptoms with elevated PD-L1 expression in LMP-1-positive cases of HL, suggesting EBV-driven immune evasion. These results underscore the importance of considering HL subtypes and EBV status when evaluating biomarkers. Incorporating EBV and PD-L1 targeted therapies could offer more effective, personalized treatment approaches, particularly for EBV-positive HL cases.

## Introduction

Hodgkin lymphoma (HL) is a hematolymphoid cancer marked by Reed-Sternberg (RS) cells in a reactive background. Its incidence varies across and within populations, showing a bimodal age distribution in young and older adults. Globally, HL accounts for 0.4% of new cancer cases and 0.2% of cancer deaths [1].

In 2022, a total of 82,469 new cases of HL were reported globally. The global age-standardized incidence rate was 0.95 per 100,000 people. In 2022, a total of 22,733 deaths of HL were recorded worldwide. The age-standardized mortality rate was 0.24 per 100,000 people [1].

The HL microenvironment is unique because RS cells make up less than 1% of the tumor, surrounded by a dense, diverse inflammatory infiltrate of T cells, B cells, granulocytes, eosinophils, and stromal cells [2]. The interaction between RS and the microenvironment's reactive cells sustains tumor growth and survival. Programmed death ligand 1 (PD-L1) is a molecule expressed on antigen-presenting cells that engages the programmed death protein 1 (PD-1) receptor on T cells and inhibits T-cell receptor signaling. The PD-1 axis can be exploited by tumor cells to dampen host anti-tumor immune responses and foster tumor cell survival [3].

The PD-L1 is a contributor to the immunosuppressive microenvironment of HL. PD-1 signaling results in “T-cell exhaustion,” i.e., T cells in HL display features of chronic ineffectual antigen encounter, a functional phenotype that can be reversed by PD-1 blockade [4]. More importantly, blocking the PD-1/PD-L1 pathway has proven to be an effective treatment approach for classical HL [5,6]. Additionally, the extent of PD-L1 expression is associated with a positive response to PD-1/PD-L1 inhibitors [7].

HL is reported to have the highest incidence of PD-L1 expression among other lymphomas, and this expression has been correlated with the presence of Epstein-Barr virus (EBV) in tumor cells. The gene products of EBV, such as latent membrane protein 1 (LMP-1), have been shown to upregulate PD-L1 expression in several patients with HL. Most patients diagnosed with HL have strong PD-L1/PD-L2 expression [3]. This study has been undertaken to see the expression of PD-L1 and EBV LMP-1 in HL and intends to explore the association of EBV and PD-L1 in various subtypes of HL and study the various clinicopathologic parameters in the PD-L1 and EBV-positive cases of HL.

## Materials and methods

The study was conducted in the Department of Pathology in collaboration with the Department of Radiation Oncology, North Eastern Indira Gandhi Regional Institute of Health and Medical Sciences (NEIGRIHMS), Shillong.

Period of study

The ambispective study period was from January 2014 to January 2024, after obtaining approval from the NEIGRIHMS Scientific Advisory Committee (NSAC) and the Institute Ethics Committee (IEC) (approval number: NEIGR/IEC/M7/T19/2022).

Study population

A total of 40 patients diagnosed with HL, who fulfilled our inclusion and exclusion criteria, were included in our study.

Inclusion and exclusion criteria

All patients diagnosed with HL by histopathological examination at NEIGRIHMS between January 2014 and January 2024 were included in the study. Patients were excluded if their tissue blocks contained inadequate material or if the blocks had been sent outside the institution for treatment-related purposes.

Study procedure

The clinical and pathological details of each case were recorded according to the proforma. Tumors were verified histologically. Hematoxylin and eosin (H&E) sections were analyzed, and the block with maximum tumor density was selected for PD-L1 and LMP-1 staining. Immunohistochemistry (IHC) was performed using monoclonal antibodies against PD-L1 and LMP-1 in HL cases and subsequently evaluated. Immunohistochemical staining of PD-L1 and LMP-1 was performed using the 3,3′-diaminobenzidine (DAB) peroxidase method, and negative/positive controls were established as recommended. Only cases that were histopathologically diagnosed as HL were selected for IHC. Interpretation of positive slides was done using a light microscope. Initially, we established a 5% PD-L1 staining threshold for RS cells and then assessed the staining quality. Tumors with more than 5% of RS cells exhibiting PD-L1 staining were designated as positive. Test results were considered positive when cytoplasmic and/or membranous staining was observed. EBV LMP-1 positivity in RS cells was determined by the presence of membranous and cytoplasmic staining, while aberrant nuclear staining was considered negative.

Statistical analysis

The data were entered into Microsoft Excel (Microsoft Corp., Redmond, WA, USA) and analyzed using IBM SPSS Statistics for Windows, Version 28 (Released 2021; IBM Corp., Armonk, New York, United States). Descriptive statistics were applied to summarize categorical variables, presented as counts and percentages, while continuous variables were expressed as means and ranges. Fisher’s exact test and the chi-square (χ^2^) test were used to evaluate the association between PD-L1 and LMP-1 expression and various clinicopathological parameters. The threshold of 0.05 for probability (p) was deemed statistically significant.

## Results

During the study period, a total of 40 cases histopathologically diagnosed as HL at our facility were included in the analysis. Patient ages ranged from 4 to 82 years, with a mean age of 32.3 years. Most patients belonged to the 21-40 year age group, with 19 patients in this category.

**Figure 1:**
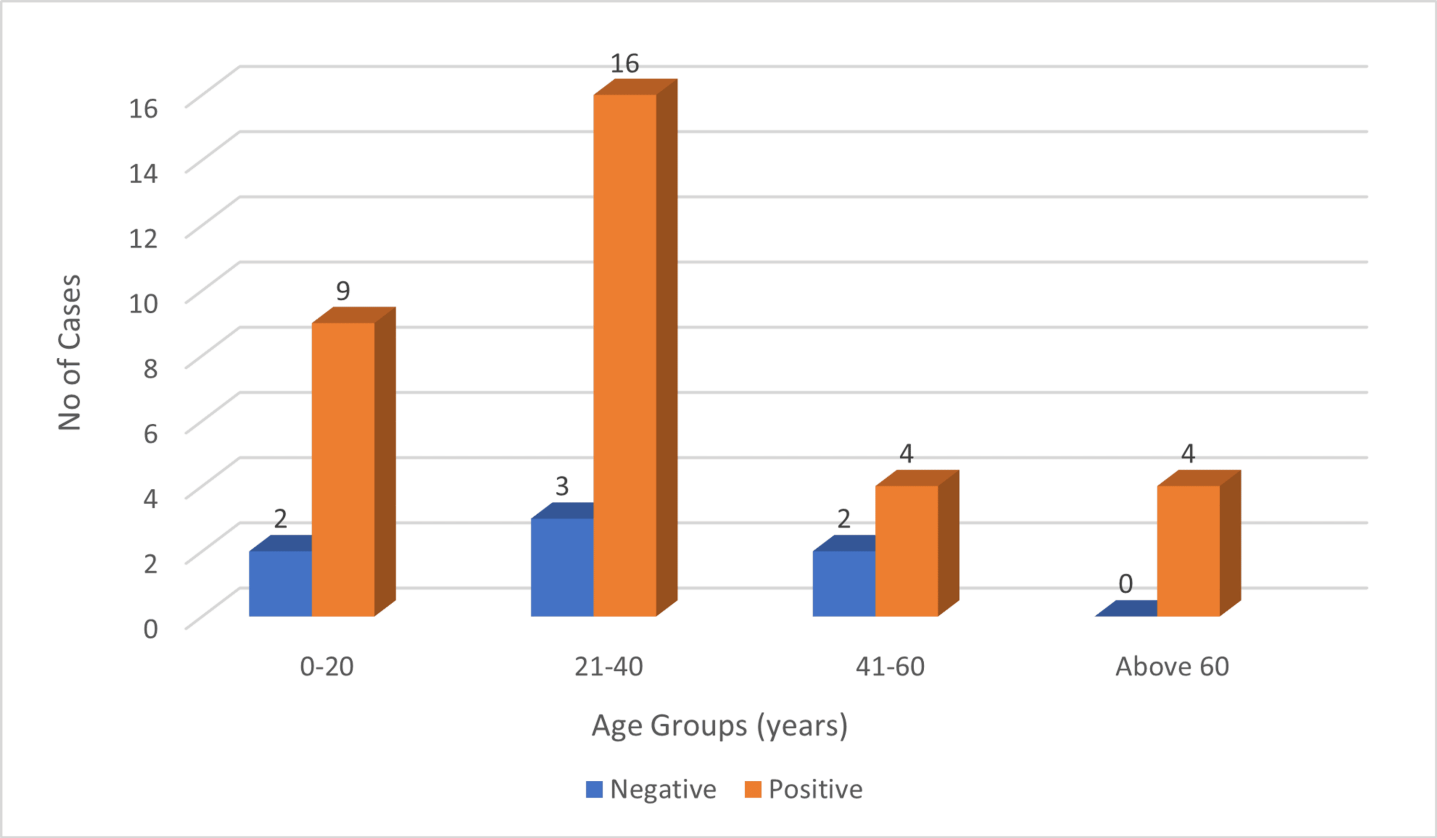
Age groups versus PD-L1 expression. PD-L1: programmed death ligand 1

**Figure 2:**
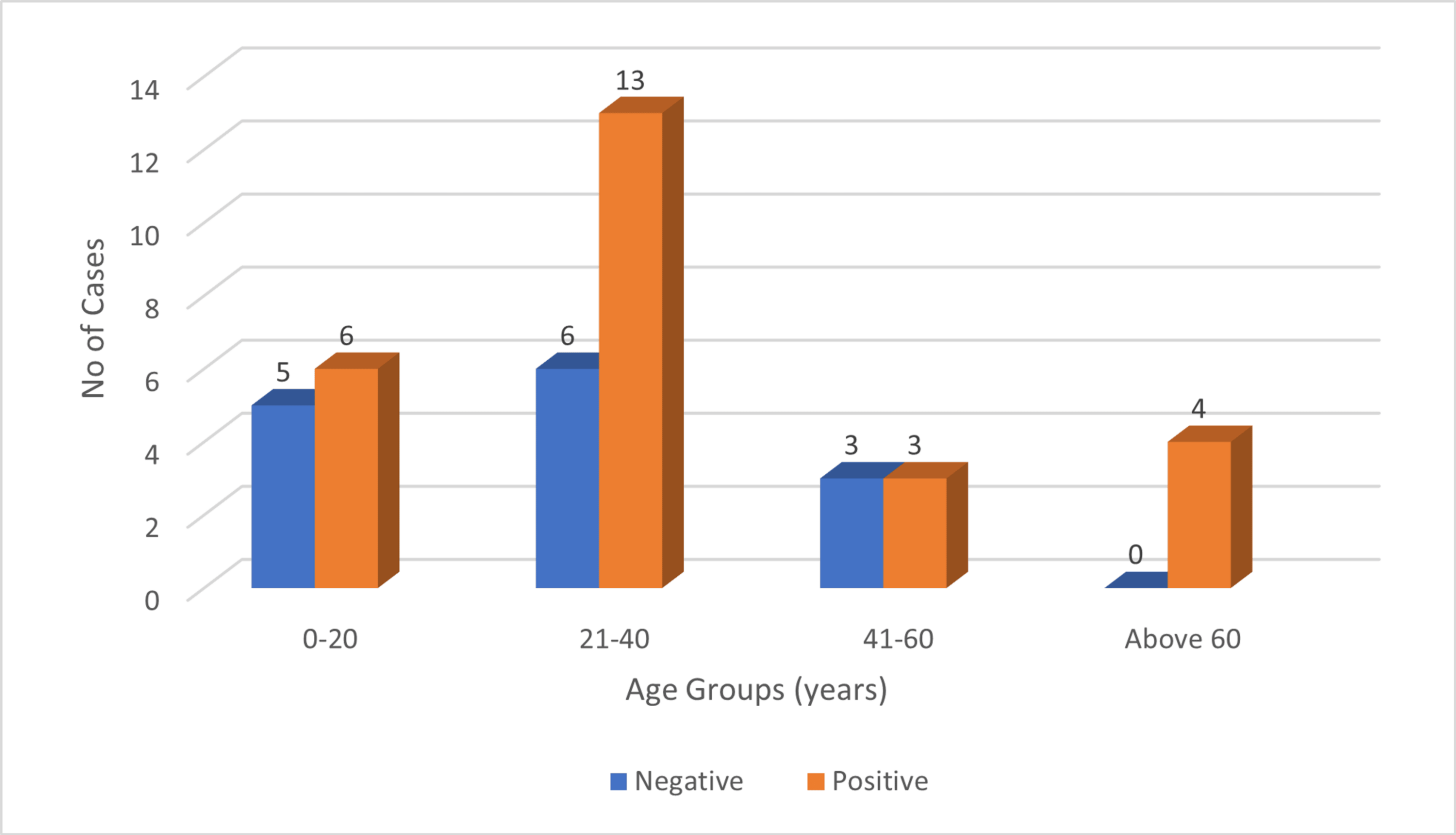
Age groups versus LMP-1 expression. LMP-1: latent membrane protein 1

PD-L1 expression was observed across all age groups, with the highest frequency in the 21-40 years group (48.48%, 16/33), followed by 0-20 years (27.28%, 9/33), 41-60 years (12.12%, 4/33), and ≥60 years (12.12%, 4/33) (Figure 1). LMP-1 expression was also seen in all age groups, most frequently in the 21-40 years group (50%, 13/26), followed by 0-20 years (23%, 6/26), ≥60 years (15.38%, 4/26), and 41-60 years (11.53%, 3/26) (Figure 2).

**Figure 3:**
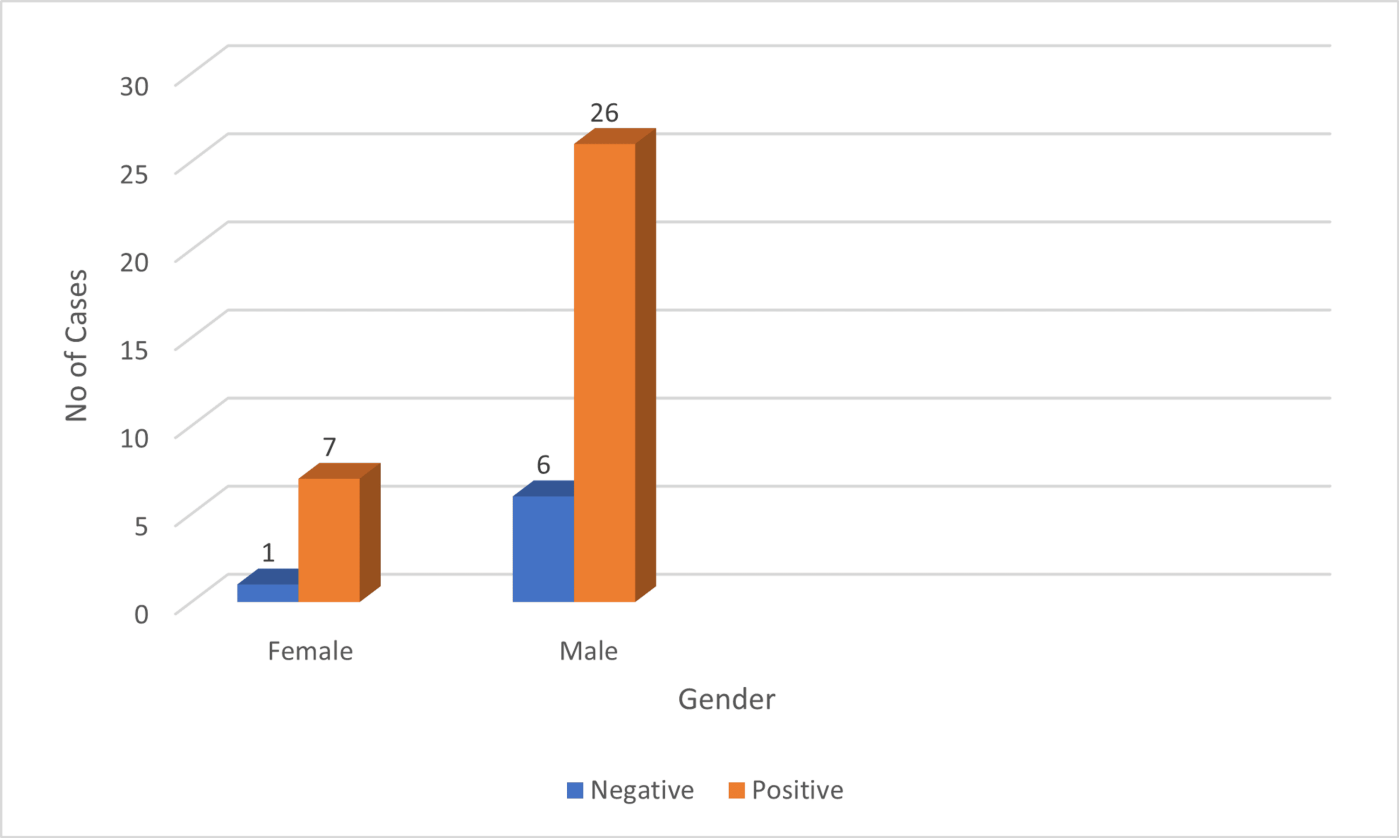
Gender versus PD-L1 expression. PD-L1: programmed death ligand 1

**Figure 4:**
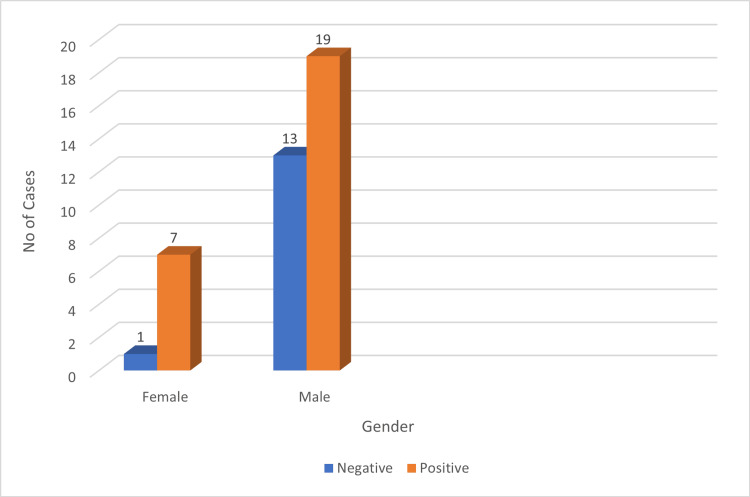
Gender versus LMP-1 expression. LMP-1: latent membrane protein 1

Among the 40 cases, males constituted the majority with 32 cases (80%), while females accounted for eight cases (20%). PD-L1 positivity in tumor cells was observed in 78.78% (26/33) of male cases and 21.21% (7/33) of female cases (Figure 3). For LMP-1 expression, 73.07% (19/26) of positive cases were male, and 26.92% (7/26) were female (Figure 4).

Of the 40 cases, 67.5% (27/40) were positive for B symptoms, while 32.5% (13/40) were negative. Among those with B symptoms, 75.75% (25/33) showed PD-L1 positivity compared to 24.25% (8/33) of those without B symptoms (Table 1). Similarly, 80.76% (21/26) of the B symptom-positive cases and 19.23% (5/26) of the B symptom-negative cases showed LMP-1 positivity (Table 2).

**Table 1:** Distribution of B symptoms versus PD-L1 expression in tumor cells. Note: B symptoms include fever, weight loss of more than 10% over six months, and night sweats. PD-L1: programmed death ligand 1

	PD-L1 expression		
B symptoms	Negative	Positive	Grand total
Negative	5	8	13
Positive	2	25	27
Grand total	7	33	40

**Table 2:** Distribution of B symptoms versus LMP-1 expression. Note: B symptoms include fever, weight loss of more than 10% over six months, and night sweats. LMP-1: latent membrane protein 1

	LMP-1 expression		
B symptoms	Negative	Positive	Grand total
Negative	8	5	13
Positive	6	21	27
Grand total	14	26	40

The majority of cases demonstrated nodal involvement, with the cervical lymph nodes being the most commonly affected site (55%, 22/40), followed by the axillary lymph nodes (20%, 8/40). Other nodal regions were less frequently involved, and no cases of extranodal involvement were observed. PD-L1 expression was detected in 82.5% (33/40) of all cases, with cervical lymph node involvement comprising the highest proportion of PD-L1-positive cases (57.57%, 19/33) (Table 3). Similarly, LMP-1 expression was observed in 65% (26/40) of the cases, with cervical lymph nodes accounting for 57.69% (15/26) of these positive cases (Table 4).

**Table 3:** Distribution of site versus PD-L1 expression in tumor cells. PD-L1: programmed death ligand 1

Site	PD-L1 result		
	Negative	Positive	Grand total
Axillary lymph node	2	6	8
Cervical lymph node	3	19	22
Inguinal node	2	4	6
Supraclavicular lymph node	0	4	4
Grand total	7	33	40

**Table 4:** Distribution of site versus LMP-1 expression. LMP-1: latent membrane protein 1

Site	LMP-1 result		
	Negative	Positive	Grand total
Axillary lymph node	3	5	8
Cervical lymph node	7	15	22
Inguinal node	2	4	6
Supraclavicular lymph node	2	2	4
Grand total	14	26	40

With respect to histological subtypes, mixed cellularity classical HL (MCCHL) was the most common, comprising 19 cases (47.5%), followed by nodular sclerosis classical HL (NSCHL) with 11 cases (27.5%), lymphocyte-depleted classical HL (LDCHL) with six cases (15%), lymphocyte-rich classical HL (LRCHL) with three cases (7.5%), and nodular lymphocyte predominant HL (NLPHL) with one case (2.5%).

PD-L1 expression in tumor cells was observed in 84.2% (16/19) of MCCHL cases, 100% (11/11) of NSCHL cases, 66.6% (4/6) of LDCHL cases, and 66.6% (2/3) of LRCHL cases. No PD-L1 expression was detected in the single case of NLPHL (Table 5).

**Table 5:** Distribution of histological subtype versus the PD-L1 expression in tumor cells. PD-L1: programmed death ligand 1

Histological diagnosis	PD-L1 result		
	Negative	Positive	Grand total
Lymphocyte-rich classical Hodgkin lymphoma	1	2	3
Lymphocyte-depleted classical Hodgkin lymphoma	2	4	6
Mixed-cellularity classical Hodgkin lymphoma	3	16	19
Nodular lymphocyte predominant Hodgkin lymphoma	1	0	1
Nodular sclerosis classical Hodgkin lymphoma	0	11	11
Grand total	7	33	40

Regarding LMP-1 expression, positivity was found in 89.4% (17/19) of MCCHL cases, 36.4% (4/11) of NSCHL cases, 50% (3/6) of LDCHL cases, and 66.6% (2/3) of LRCHL cases. Similar to PD-L1, no LMP-1 expression was observed in the NLPHL case (Table 6).

**Table 6:** Distribution of histological subtype versus the LMP-1 expression. LMP-1: latent membrane protein 1

Histological diagnosis	LMP-1 result		
	Negative	Positive	Grand total
Lymphocyte-rich classical Hodgkin lymphoma	1	2	3
Lymphocyte-depleted classical Hodgkin lymphoma	3	3	6
Mixed-cellularity classical Hodgkin lymphoma	2	17	19
Nodular lymphocyte predominant Hodgkin lymphoma	1	0	1
Nodular sclerosis classical Hodgkin lymphoma	7	4	11
Grand total	14	26	40

EBV positivity was detected in 22 of 33 (66.7%) PD-L1-positive cases compared to four out of seven (57.1%) PD-L1-negative cases; however, this difference was not statistically significant (p = 0.67).

Bivariate analysis (Table 7) revealed that the presence of B symptoms and the histological subtype of HL showed a statistically significant association with LMP-1 expression (p < 0.05). Similarly, B symptoms demonstrated a significant association with PD-L1 expression (p < 0.05). However, no significant correlation was observed between PD-L1 or LMP-1 expression and age, gender, or site. Additionally, no significant association was found between PD-L1 and LMP-1 expression in the cases analyzed in this study.

**Table 7:** Significance of each parameter in determining PD-L1 and LMP-1 expression observed using Fisher's exact test and chi-squared test with simulated p-value. PD-L1: programmed death ligand 1; LMP-1: latent membrane protein 1

Variables	PD-L1	LMP-1
Age range	0.58	0.33
Gender	0.07	0.13
B symptoms	0.015	0.014
Site	0.48	0.91
Histological subtype	0.07	0.022

**Figure 5:**
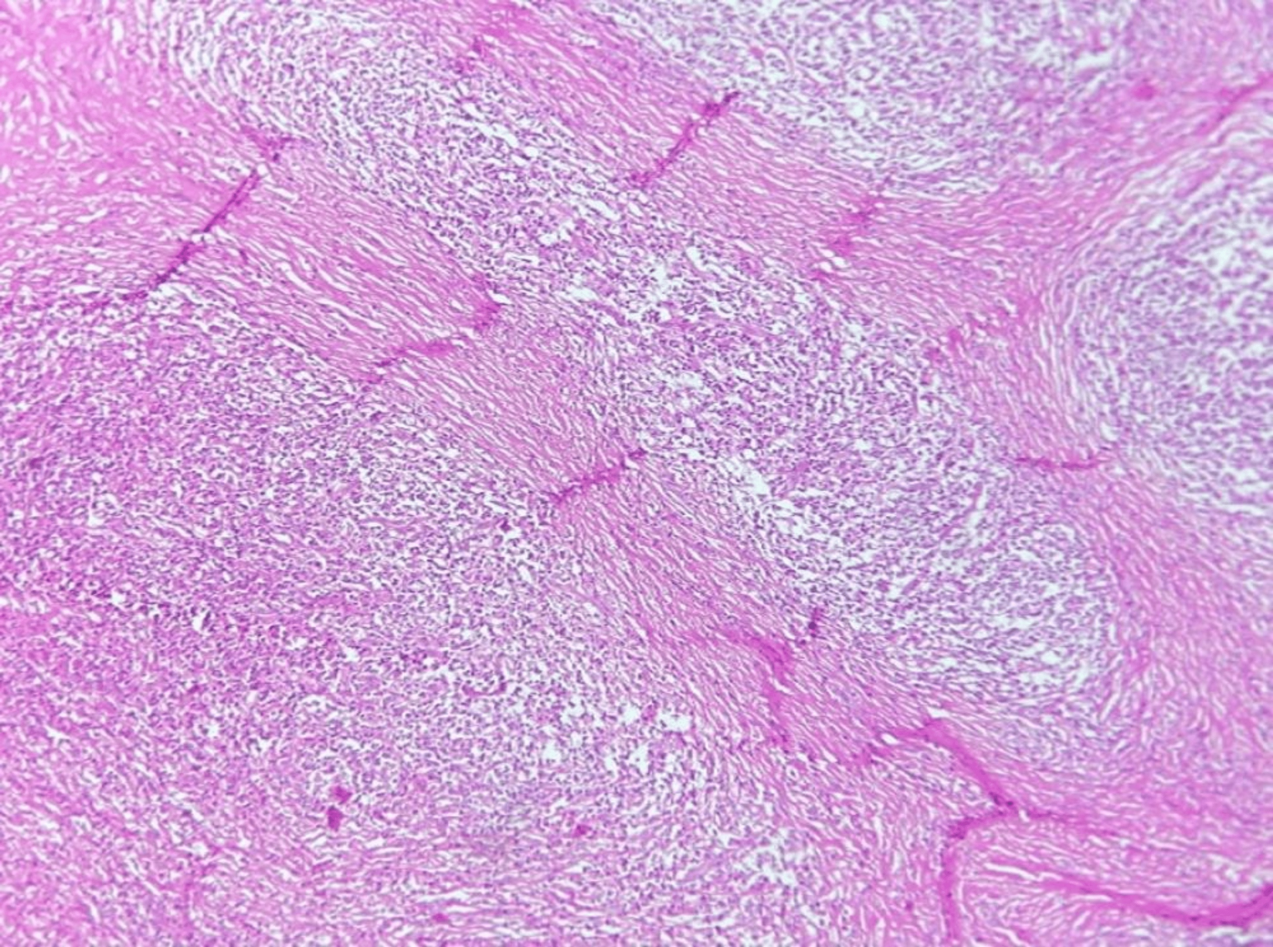
Nodular sclerosis Hodgkin lymphoma. Hematoxylin and eosin (H&E) stain at 4× magnification shows thick fibrous collagen bands dividing the lymph node into distinct nodules.

**Figure 6:**
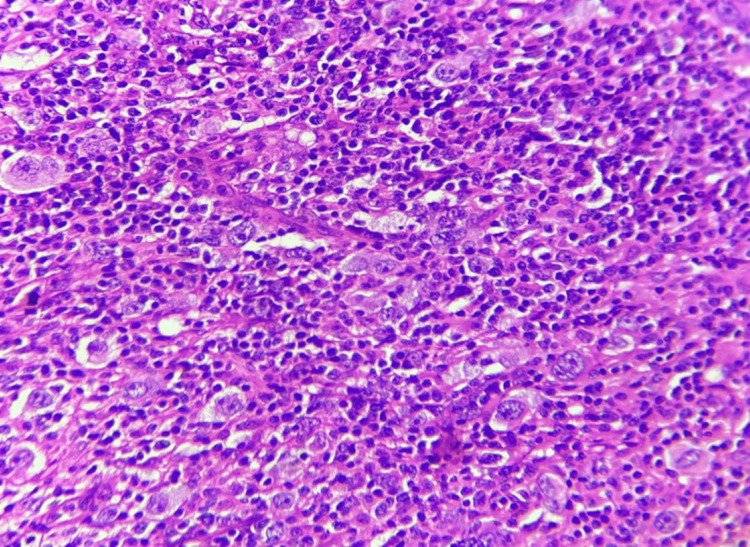
Lymphocyte rich Hodgkin lymphoma. Hematoxylin and eosin (H&E) stain at 10× magnification shows Reed-Sternberg cells scattered within a background rich in small lymphocytes.

**Figure 7:**
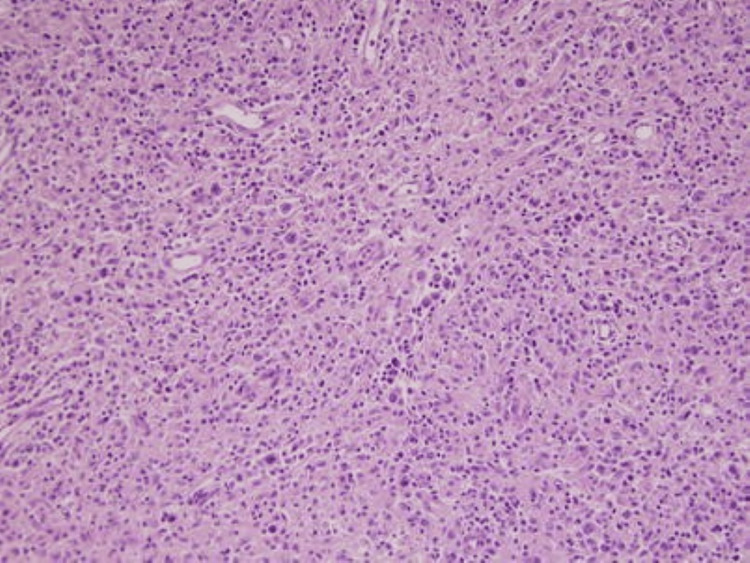
Lymphocyte depleted Hodgkin lymphoma. Hematoxylin and eosin (H&E) at 10x shows numerous Reed-Sternberg cells with sparse lymphocytes.

**Figure 8:**
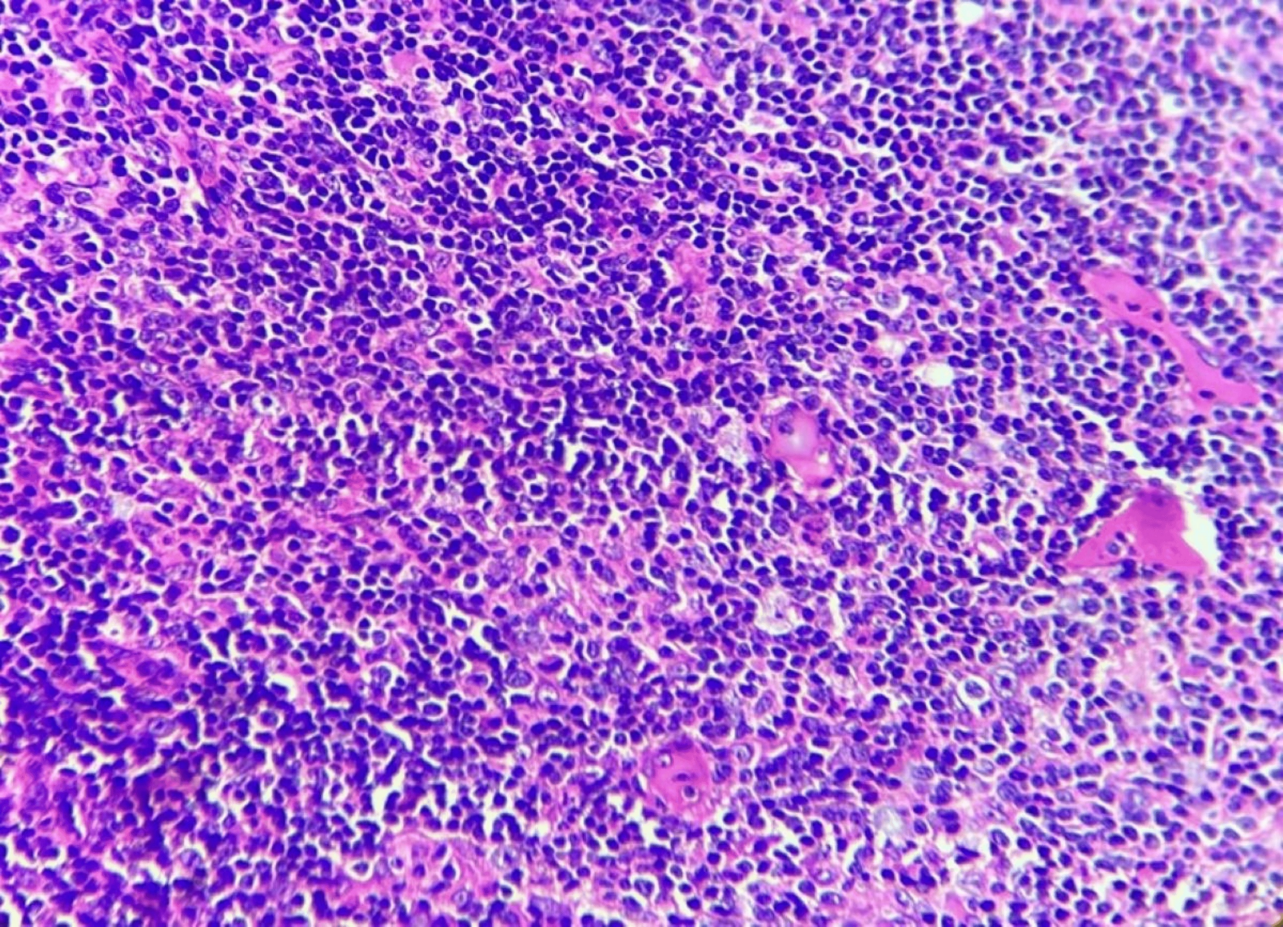
Nodular lymphocyte predominant Hodgkin lymphoma. Hematoxylin and eosin (H&E) at 10x shows lymphocytic and histiocytic (LP, “popcorn”) cells within a nodular background rich in small lymphocytes.

**Figure 9:**
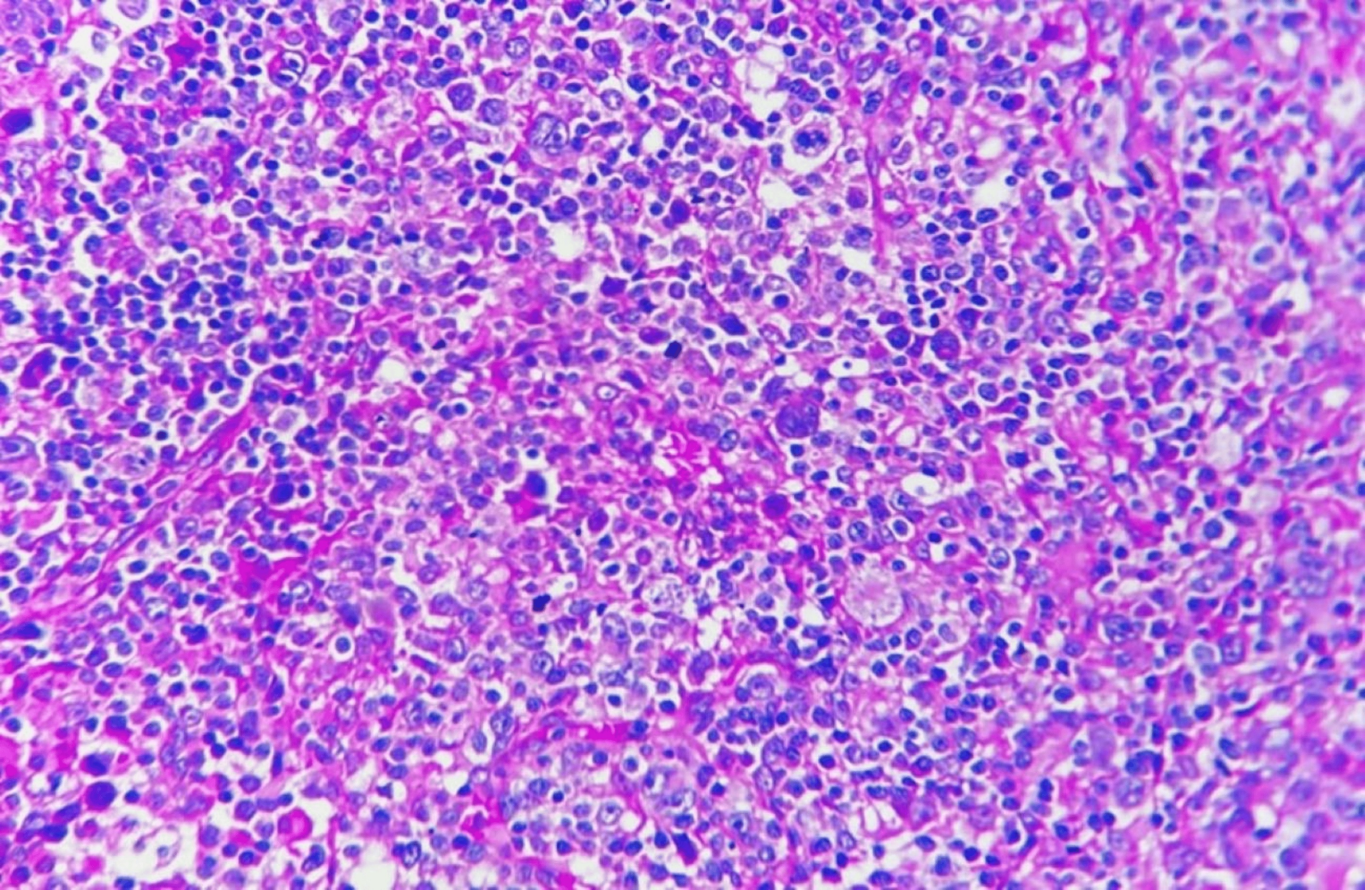
Mixed cellularity Hodgkin lymphoma. Hematoxylin and eosin (H&E) stain at 10× magnification shows Reed-Sternberg cells within a polymorphous cellular background.

**Figure 10:**
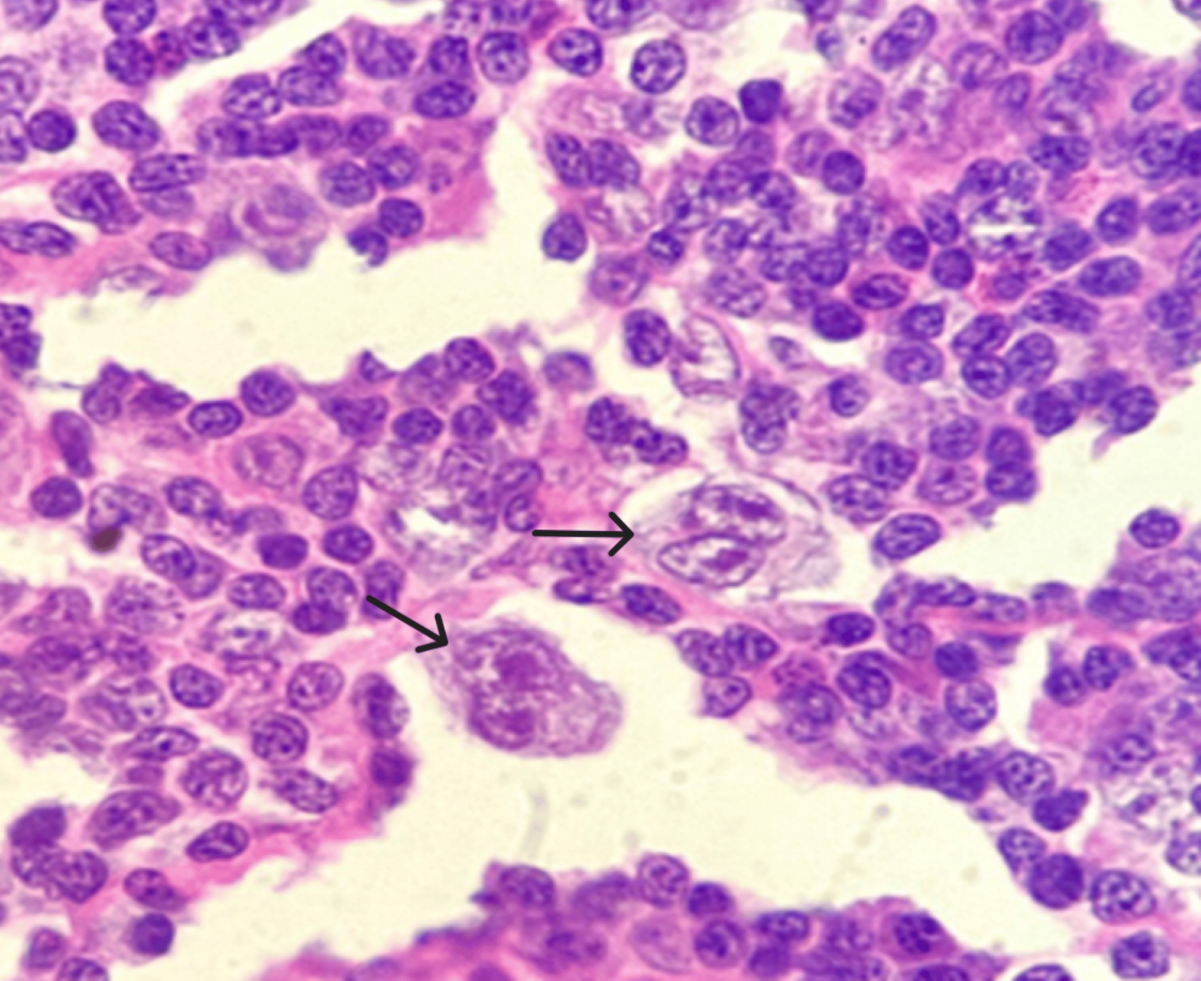
Reed-Sternberg cells (arrows) (H&E, 40×). H&E: hematoxylin and eosin

**Figure 11:**
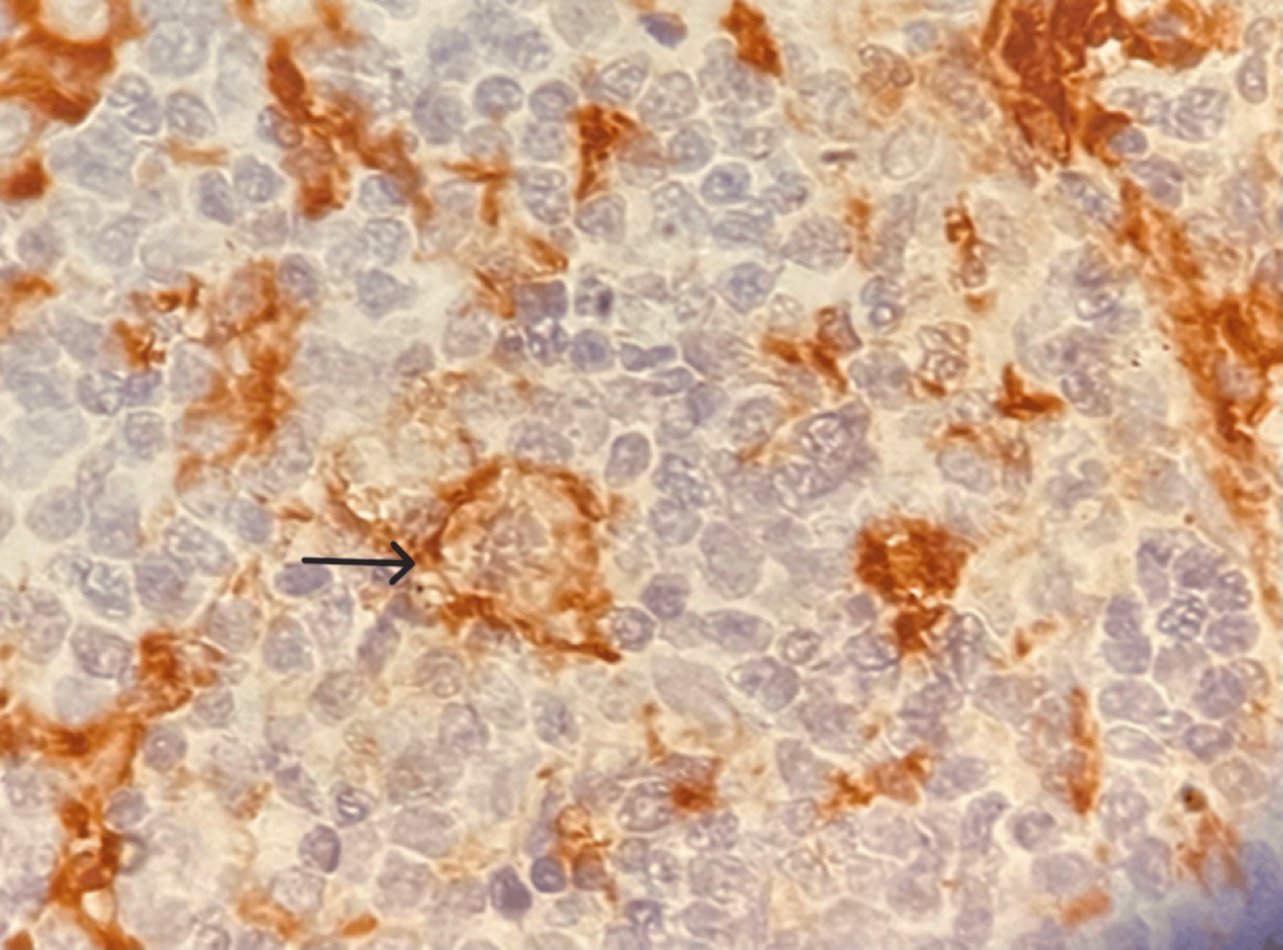
Immunohistochemistry (IHC), 40× magnification. Membranous PD-L1 expression is highlighted in Reed-Sternberg cells (arrows). PD-L1: programmed death ligand 1

**Figure 12:**
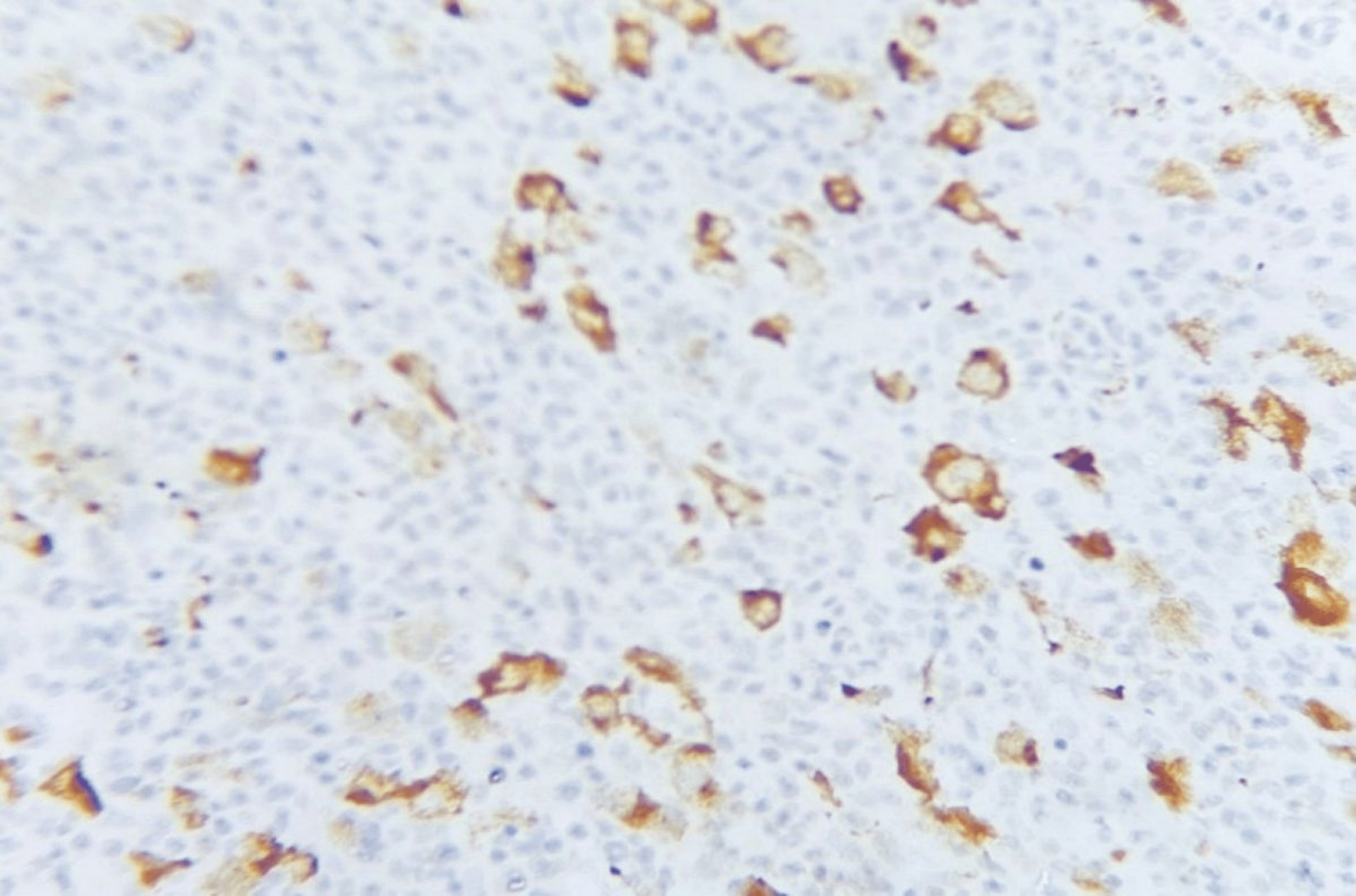
Immunohistochemistry (IHC), 40× magnification. LMP-1 expression in Reed-Sternberg cells showing cytoplasmic and membranous staining, with an occasional cap-like staining pattern. LMP-1: latent membrane protein 1

Figures 5-10 show the histopathological subtypes of HL with H&E staining, including nodular sclerosis (Figure 5), lymphocyte-rich (Figure 6), lymphocyte-depleted (Figure 7), NLPHL (Figure 8), mixed cellularity (Figure 9), and RS cells (Figure 10). Immunostaining in Figures 11-12 demonstrates PD-L1 and LMP-1 expression, respectively, in RS cells.

## Discussion

A total of 40 cases histopathologically diagnosed as HL in our facility were included in the analysis. We found that the majority of the HL cases were in the age group 4-82 years (mean age: 32.3 years) with a male preponderance (80%). The study done by Özdemir et al. showed male preponderance (69.6%) with a median age of 26 years and an age range of 3-85 years [8].

A similar study done by Karnik et al. showed male preponderance (73%), and subjects were in the age range of 3-65 years [9]. In another study done by Ozturk et al., a similar male preponderance (64%) is seen, and in the range of 20-75 years with a median age of 43 years [10]. Also, the study done by Hashmi et al. showed a similar male preponderance, and the mean age of patients was 35.11 years [11]. However, a study done by Jung et al. showed female preponderance (M:F = 2:3) with a median age of 41 years and an age range of 27-72 years [12].

The statistical analysis conducted on age and gender did not reveal any significant association with PD-L1 positivity and EBV LMP-1 positivity, as indicated by a p-value greater than 0.05. This finding is consistent with the study conducted by Ozturk et al., which also found no significant correlation between age and the positivity of PD-L1 (p-value = 1) and EBV (p-value = 0.51) [10]. Similarly, another study by Özdemir et al. corroborated these results, demonstrating no significant association between PD-L1 positivity and both age and gender [8].

HL is known to have a bimodal age distribution, with peaks in young adulthood and later in life, which may explain the wide age range observed in these studies. The male predominance seen across these studies could be attributed to a combination of genetic, hormonal, and environmental factors that may differentially affect males and females. Additionally, social and healthcare access factors might contribute to the observed demographics, as males might be more likely to seek medical attention or be diagnosed at certain stages of the disease. These consistent findings across multiple studies highlight the importance of considering demographic factors in the understanding and management of HL.

B symptoms

In the current study, a notable 67.5% of patients were found to exhibit B symptoms (Table 8). Among these patients, 25 cases demonstrated PD-L1 positivity, while 21 cases were positive for LMP-1. Importantly, a significant correlation was identified between the presence of B symptoms and both PD-L1 positivity (p-value = 0.015) and LMP-1 positivity (p-value = 0.014). This finding aligns with the research conducted by Ozturk et al., where 52.8% of patients were positive for B symptoms. In their study, a significant correlation was also found between B symptoms and PD-L1 positivity (p-value = 0.008), as well as between B symptoms and EBV positivity (p-value = 0.007) [10]. Similarly, a study done by Hollander et al. showed 52% PD-L1 positivity for B symptoms, and with a significant correlation, as the p-value is 0.02 [13]. Also, a study by Özdemir et al. reported a positivity rate for B symptoms of 51.3% [8]. However, in this study, no significant correlation could be established between the presence of B symptoms and PD-L1 positivity, which is in concordance with our study. Overall, the results of the present study are in agreement with the findings of previous research, underscoring the potential significance of B symptoms in relation to PD-L1 and LMP-1 positivity. This consistent correlation across multiple studies suggests that further investigation into the mechanisms underlying these associations is warranted.

**Table 8:** Correlation between B symptoms with PD-L1 and LMP-1 expression across various studies. PD-L1: programmed death ligand 1; LMP-1: latent membrane protein 1; EBV: Epstein-Barr virus

Parameters	Özdemir et al. [8]	Ozturk et al. [10]	Hollander et al. [13]	Present Study
B symptoms	51.3%	52.8%	48%	67.5%
p-value with PD-L1 positivity	0.66	0.008	0.02	0.015
p-value with EBV positivity	N/A	0.007	N/A	0.014

Site

The present study revealed that HL most frequently occurs in lymph nodes, particularly the cervical nodes, with a 55% incidence. Among these cases, 19 showed PD-L1 positivity, and 15 showed LMP-1 positivity. A similar study by Hashmi et al. found a higher rate of nodal involvement (57 out of 66 cases, or 86.36%) compared to extranodal involvement (9 out of 66 cases, or 13.63%) [11]. Additionally, research by Sherief et al. indicated that 96.6% of all cases involved nodal sites, with the cervical nodes being the most common site at 51.8% [14]. However, in a research conducted by Jung et al., only 39% of regional lymph nodes were involved [12]. Statistical analysis for PD-L1 and LMP-1 expression in different sites did not show any significant association, with a p-value greater than 0.05.

Histological subtypes

There are various histologic subtypes of HL known and classified based on their morphology, architecture, background non-malignant lymphoid population, presence of fibrosis, relative percentage of Hodgkin and Reed-Sternberg (HRS), and immunophenotype. In high-income nations like the USA, NSCHL is the most frequent subtype of HL [15]. On the other hand, MCCHL is more frequent in low-income nations. The most common histological subtype observed in our study was MCCHL at 47.5% followed by NSCHL at 27.5%, LDCHL at 15%, LRCHL at 7.5%, and NLPHL at 2.5%.

Similar studies done by Hashmi et al. found MCCHL to be the most common subtype, accounting for 34.8% of cases (23/66) [11], and a study done by Barakzai and Pervez showed nearly 47 cases (51%) being of the MCCHL subtype, 40 cases (44%) were NSCHL and four cases (4.4%) of LRCHL [16]. While that done by Karnik et al. showed NSCHL as the most frequent histological subtype (50%), followed by MCCHL (38%), LDCHL (8%), and NLPHL (4%) (Table 9) [9]. In our study of 40 HL cases, LMP-1 was positive in 26 cases, with the highest in MCCHL (89.4%), and lowest in NLPHL(0%). These findings align with Hashmi et al. [11] and Özdemir et al. [8], emphasizing significant LMP-1 expression in MCCHL. Also, Pinkus et al. [17] and Weinreb et al. [18] report lower LMP-1 positivity in the NLPHL subtype. However, Karnik et al. found higher LMP-1 expression in NSCHL compared to MCCHL, indicating variability in LMP-1 positivity across different HL subtypes (Table 9) [9]. Furthermore, bivariate analysis revealed a significant correlation of LMP-1 expression with histological subtypes (p-value = 0.022), consistent with findings from studies by Weinreb et al. [19] and Özdemir et al. [8].

**Table 9:** EBV positivity (%) with various histological subtypes along with their p-values. EBV: Epstein-Barr virus; MCCHL: mixed cellularity classical Hodgkin lymphoma; NSCHL: nodular sclerosis classical Hodgkin lymphoma; LRCHL: lymphocyte-rich classical Hodgkin lymphoma; LDCHL: lymphocyte-depleted classical Hodgkin lymphoma; NLPHL: nodular lymphocyte-predominant Hodgkin lymphoma

Subtypes	Karnik et al. [9]	Weinreb et al. [18]	Pinkus et al. [17]	Hashmi et al. [11]	Weinreb et al. [19]	Özdemir et al. [8]	Present study
MCCHL	79%	85%	69%	73.9%	N/A	69%	89.4%
NSCHL	86%	39%	14%	66.7%	N/A	32%	36.4%
LRCHL	N/A	N/A	N/A	40%	N/A	100%	66.6%
LDCHL	75%	50%	66.67%	66.7%	N/A	0%	50%
NLPHL	N/A	31%	0%	N/A	N/A	N/A	0%
p-value	N/A	N/A	N/A	0.65	0.005	0.01	0.022

The present study demonstrated PDL1 positivity in 82.5% of cases, a result that closely aligns with the findings of Özdemir et al. [8] (89.2%) and Chen et al. (87%) [20], indicating consistency among these studies. In contrast, lower rates reported by Menter et al. [21] (70%) and Hollander et al. (72.3%) [13] suggest variability that may stem from differences in sample characteristics, methodologies, or measurement techniques. These discrepancies emphasize the influence of study-specific factors on outcomes and highlight the need for standardized approaches in future research.

PD-L1 positivity with various subtypes of HL

Out of the 40 HL cases analyzed, 33 cases (82.5%) showed positive PD-L1 expression. Among the subtypes, positivity was observed in 16 cases (84.2%) of MCCHL, 11 cases (100%) of NSCHL, four cases (66.6%) of LDCHL, and two cases (66.6%) of LRCHL. None of the NLPHL cases demonstrated PD-L1 positivity in tumor cells. However, no significant association is noted between various subtypes of HL with PD-L1 positivity.

This result was comparable to studies conducted by Hollander et al., where PD-L1 expression was found to be maximum in the nodular sclerosis subtype (78%), followed by the mixed cellularity subtype (20%), and only 3% in the lymphocyte-rich + lymphocyte-depleted + unclassifiable subtype of HL [13]. Chen et al. showed PD-L1 expression in 88% of MCCHL, 84% of NSCHL, and only 13.34% of NLPHL cases [20]. Similarly, a study by Green et al. showed PD-L1 positivity only in primary cases of NSCHL, with no PD-L1 expression observed in MCCHL cases [22]. These findings highlight the variability in PD-L1 expression among different subtypes of HL, underscoring the importance of subtype-specific approaches in both research and treatment strategies.

Comparison of PD-L1 positivity according to EBV positivity

Our study found that 84.6% (22 out of 26) of LMP-1-positive cases were also PD-L1 positive, indicating a notable prevalence of PD-L1 expression in cases associated with EBV. Although this correlation did not reach statistical significance (p-value = 0.67), the higher percentage of PD-L1 expression in LMP-1-positive cases suggests an important trend where EBV may influence PD-L1 upregulation in HL. This could have significant implications for understanding the tumor microenvironment and developing targeted therapies. However, our inability to establish a definitive correlation between PD-L1 and EBV positivity may be due to the use of LMP-1 as a marker for EBV detection instead of the more specific Epstein-Barr virus-encoded RNA (EBER) used in other studies. LMP-1, while indicative of EBV presence, may not be as sensitive or specific as EBER in identifying EBV infection in HL cases. This methodological difference could explain why our findings did not align with the statistically significant correlations reported in other research, underscoring the importance of marker selection in studying the relationship between PD-L1 expression and EBV positivity.

Our findings align with Özdemir et al., where a higher percentage (89.6%, 26 out of 29) of LMP-1 positive cases also tested positive for PD-L1 [8]. However, no significant correlation was observed between PD-L1 and LMP-1 positivity (p-value > 0.05). In contrast, Ozturk et al. found a significant association between PD-L1 positivity in RS cells and EBV positivity (p < 0.05) [10].

A study by Green et al. reported that EBV-positive HL cases exhibit higher LMP-1 and PD-L1 expression, supporting the role of EBV in modulating the tumor microenvironment and promoting immune evasion [22]. Similarly, another study by Chen et al. highlighted the correlation between EBV status and PD-L1 expression in HL [20]. A total of 92% (11 of 12) of EBV-positive HL cases exhibited strong membranous PD-L1 staining in malignant RS cells, while one EBV-positive NSCHL case showed weak staining in 90% of RS cells. Among EBV-negative cases, 69% (20 of 29) were positive for PD-L1.

PD-L1 is a key immune checkpoint protein that, when expressed on the surface of tumor cells, interacts with the PD-1 receptor on T cells, leading to immune suppression and allowing tumor cells to evade immune detection. The increased PD-L1 expression in EBV-positive HL cases suggests a potential mechanism by which EBV-associated tumors might escape immune surveillance, thereby contributing to disease progression. Our findings are consistent with those of other studies, which have reported higher PD-L1 expression in EBV-positive HL, emphasizing the potential therapeutic implications of targeting the PD-1/PD-L1 axis in these patients.

The significance of the test results in bivariate analysis indicates that LMP-1 expression correlates significantly with B symptoms and the histological subtype of HL, with a p-value of <0.05. However, this study did not find any correlation between LMP-1 expression and age, gender, or site, as the p-values for these variables were > 0.05. Similarly, PD-L1 expression shows a significant correlation with B symptoms. However, there was no significant correlation observed with age, gender, site, histological subtypes, or EBV expression.

Limitations

The limited sample size of just 40 cases might constrain the broader applicability of the findings. Additionally, using LMP-1 instead of more precise markers like EBER for detecting EBV could impact the accuracy of the results. The expression levels of PD-L1 and LMP-1 also varied significantly across different HL subtypes, which might affect interpretations. Missing staging data hindered full disease evaluation. The study's design restricts our ability to conclusively determine the effects of PD-L1 and LMP-1 on patient outcomes such as prognosis or treatment response. Although some associations between symptoms and marker positivity were found, these were mostly in basic comparisons; more comprehensive analyses or larger studies are needed to verify these links. Furthermore, the findings are region-specific and may not be widely applicable due to differences in genetics, environment, and healthcare access. Addressing these limitations could improve future research and our understanding of HL.

## Conclusions

This study demonstrates that a substantial proportion of HL cases exhibit PD-L1 positivity in RS cells and the tumor microenvironment, with particularly pronounced expression in NSCHL. Conversely, LMP-1 positivity is predominantly observed in MCCHL. These findings are consistent with previous research, underscoring the variability among HL subtypes. Our study is one of the first of its kind in the Northeast region and among the few in India to explore the correlation between PD-L1 and LMP-1 expression, marking a pioneering effort with outcomes comparable to similar studies conducted elsewhere in India and globally. Furthermore, our data reveal that RS PD-L1-positive and EBV LMP-1-positive cases frequently present with B symptoms, and a higher incidence of PD-L1 expression is observed in LMP-1-positive cases. This suggests that EBV may drive PD-L1 upregulation, facilitating immune evasion in HL. These insights highlight the critical importance of considering HL subtypes and EBV status when evaluating these markers, which could serve as potential therapeutic targets. Subtype-specific, EBV-targeted, and PD-L1-targeted strategies could significantly improve treatment outcomes, particularly in EBV-positive HL cases.
